# Voltammetric Sensor Based on the Combination of Tin and Cerium Dioxide Nanoparticles with Surfactants for Quantification of Sunset Yellow FCF

**DOI:** 10.3390/s24030930

**Published:** 2024-01-31

**Authors:** Liliya Gimadutdinova, Guzel Ziyatdinova, Rustam Davletshin

**Affiliations:** 1 Analytical Chemistry Department, Kazan Federal University, Kremleyevskaya 18, Kazan 420008, Russia; liliya.gimadutdinova@gmail.com; 2 Department of High Molecular and Organoelement Compounds, Kazan Federal University, Kremleyevskaya 18, Kazan 420008, Russia; rustrdavletshin@kpfu.ru

**Keywords:** electrochemical sensors, chemically modified electrodes, metal oxide nanoparticles, surfactants, food colorants, azo dyes

## Abstract

Sunset Yellow FCF (SY FCF) is one of the widely used synthetic azo dyes in the food industry whose content has to be controlled for safety reasons. Electrochemical sensors are a promising tool for this type of task. A voltammetric sensor based on a combination of tin and cerium dioxide nanoparticles (SnO_2_–CeO_2_ NPs) with surfactants has been developed for SY FCF determination. The synergetic effect of both types of NPs has been confirmed. Surfactants of various natures (sodium lauryl sulfate (SLS), Brij^®^ 35, and hexadecylpyridinium bromide (HDPB)) have been tested as dispersive media. The best effects, i.e., the highest oxidation currents of SY FCF, have been observed in the case of HDPB. The sensor demonstrates a 4.5-fold-higher electroactive surface area and a 38-fold-higher electron transfer rate compared to the bare glassy carbon electrode (GCE). The electrooxidation of SY FCF is an irreversible, two-electron, diffusion-driven process involving proton transfer. In differential pulse mode in Britton–Robinson buffer (BRB) pH 2.0, the sensor gives a linear response to SY FCF from 0.010 to 1.0 μM and from 1.0 to 100 μM with an 8.0 nM detection limit. The absence of an interferent effect from other typical food components and colorants has been shown. The sensor has been tested on soft drinks and validated with the standard chromatographic method.

## 1. Introduction

**Figure 1:**
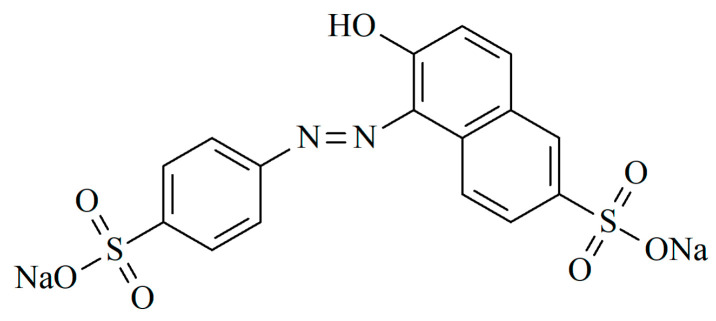
SY FCF structure.

In recent decades, synthetic dyes have become widespread in the food industry. The ability to obtain a product with a bright, uniform, easily reproducible color and to maintain a stable color for a long time under environmental conditions are the main advantages of synthetic dyes over natural ones. Low cost combined with high stability to light, oxygen, temperature, and pH changes makes synthetic dyes attractive to food manufacturers [1]. One of the most common synthetic dyes is Sunset Yellow FCF (SY FCF), which belongs to the azo food dyes group (Figure 1). When used in food and beverages, it is denoted as FD&C Yellow 6 in the United States and as E110 in Europe and Asia.

SY FCF is used as a yellow or orange dye and can also be used to produce red, brown and green colors when combined with other dyes. However, despite its beautiful color and beneficial characteristics, this dye also has negative effects. With excessive consumption, SY FCF may cause hyperactivity (especially in children), allergic reactions, asthma attacks, nausea, and vomiting [2,3]. There is also evidence of potential reproductive toxicity and immunotoxicity [4]. The daily intake dose of SY FCF is 4 mg/kg_bw_ [5]. Due to the mentioned reasons, the control of SY FCF contents in foodstuff is of practical importance.

In recent years, various methods for the detection and determination of SY FCF [6,7], such as high-performance liquid chromatography (HPLC) with UV-Vis [8,9,10] and mass-spectrometric detection [11,12], thin-layer chromatography (TLC) [13,14], capillary electrophoresis [15], spectrophotometry [16,17,18] and near-infrared spectroscopy [19], and enzyme- and quantum dot-linked immune sorbent-based assays [20,21], have been established.

However, it is important to note the inherent disadvantages of these methods, such as complicated and time-consuming sample pretreatment, expensive and bulky equipment, the need for highly qualified stuff, high cost, and the long duration of the analysis. Spectrophotometry and near-infrared spectroscopy are free of these problems but show low selectivity, limiting their practical application. Electrochemical sensors could be a worthy alternative because SY FCF is electroactive [22]. Nevertheless, electroanalytical approaches have some disadvantages, such as their relatively high detection limit and/or the insufficient sensitivity and selectivity of target analyte determination. These disadvantages can be overcome by the creation of chemically modified electrodes.

Among a great variety of modifiers, attention has been paid to transition metal oxide nanomaterials with semiconductor properties, particularly cerium(IV), zinc(II), tin(IV), titanium(IV), cadmium(II), manganese(IV), indium(III), iron(II,III), and vanadium(V) oxides [23,24,25]. Their effectivity as electrode surface modifiers for electrochemical and bio sensing has been shown on examples from a wide range of danalytes such as antioxidants [26,27,28,29], ecotoxicants [30,31,32,33], pharmaceuticals [34,35,36,37], and biomarkers [38,39,40]. The combination of various metal oxide nanomaterials provides the synergetic effect of modifiers, improvement of the analytical characteristics of target compounds, and selectivity in their determination [31,37,41,42].

Recently, transition metal oxide nanoparticles (NPs) have been shown to be an effective sensing layer for food colorants [24,43,44,45]. Among them, to date, a number of electrodes based on nano-sized metal oxides have been reported for the determination of SY FCF in various foods and pharmaceutical dosage forms. Their figures of merit are summarized in Table 1.

In a number of cases, simultaneous detection of SY FCF with tartrazine [46,47,49,50,54,55,56,58] has been achieved. However, the analytical characteristics and detection sensitivity can be further improved. To solve this problem, a mixture of transition metal oxide NPs in combination with surfactants applied as an electrode surface modifier could be considered.

The current work deals with the development of a novel voltammetric sensor based on the combination of tin and cerium dioxide NPs with surfactants for SY FCF quantification. This type of modifier is novel for SY FCF. The effect of the surfactants as dispersive media for metal oxide NPs has been studied via voltammetry and scanning electron microscopy. Cationic hexadecylpyridium bromide (HDPB) has provided the best colorant response. The sensor characteristics are improved compared to existing electrochemical methods.

## 2. Materials and Methods

### 2.1. Chemicals

The stock solution of SY FCF (10 mM) was prepared in distilled water using 98% purity reagent from Aldrich (Steinheim, Germany). Other reagents (85% tartrazine from Sigma (St. Louis, MO, USA), 85% brilliant blue FCF from Sigma-Aldrich (Steinheim, Germany), 99% ascorbic acid, 99% vanillin, 99% sorbic acid, 98% riboflavin from Aldrich (Steinheim, Germany), and carminic acid Sigma-Aldrich AG (Buchs, Switzerland), glucose, sucrose, and rhamnose of chemically pure grade from Panreac (Barcelona, Spain)) were used in the selectivity study. Their 10 mM stock solutions were prepared via dissolution of the exact weight in distilled water (ethanol rectificate for the sorbic acid). Less concentrated solutions were obtained by the exact dilution.

Commercially available metal oxide NPs were used as an electrode surface modifier. Cerium dioxide NPs water dispersion (10% wt., particle size ˂ 25 nm) from Sigma-Aldrich (St. Louis, MO, USA) and tin dioxide NPs powder (ø < 100 nm) from Aldrich (Steinheim, Germany) were used. Their 1.0 mg mL^−1^ dispersion in distilled water or surfactant medium was prepared via 10 min sonication in an ultrasonic bath WiseClean WUC-A03H (DAIHAN Scientific Co., Ltd., Wonju-si, Republic of Korea). Cerium and tin dioxide NPs were mixed in the Eppendorf, and the volume was adjusted with the corresponding solvent. Surfactant water solutions with 1.0 mM concentration were prepared from sodium lauryl sulfate (SLS) (Ph. Eur. Grade, Panreac (Barcelona, Spain), Brij^®^ 35 (98%, Acros Organics (Geel, Belgium)), and HDPB (98%, Aldrich (Steinheim, Germany)). Concentration of surfactant in the final dispersion was 0.10 mM.

### 2.2. Apparatus

Voltammetric and chronoamperometric measurements were conducted on a potentiostat/galvanostat μAutolab Type III (Eco Chemie B.V., Utrecht, The Netherlands) and NOVA 1.7.8 software connected with a 10 mL glass cell. Electrochemical impedance spectroscopy was carried out on a potentiostat/galvanostat PGSTAT 302N with FRA 32M module (Metrohm B.V., Utrecht, The Netherlands) and with NOVA 1.10.1.9 software. A glassy carbon electrode (GCE) with a 3 mm diameter (CH Instruments, Inc., Bee Cave, TX, USA) or MO_2_-NPs-modified GCE, a Ag|AgCl| KCl (sat.) electrode, and a platinum electrode were used as working, reference, and auxiliary electrodes, respectively.

The pH was measured on the “Expert-001” pH meter (Econix-Expert Ltd., Moscow, Russia) with a glassy electrode.

A MerlinTM high-resolution field emission scanning electron microscope (Carl Zeiss, Oberkochen, Germany), operated at 5 kV accelerating voltage and a 300 pA emission current, was applied for the morphological studies of the electrode surface.

HPLC was conducted on the Knauer Smartline HPLC system with a diode-array detector (Knauer, Berlin, Germany). The separation was achieved on the Agilent Zorbax SB-C18 (150 × 4.6 mm, 5 μm) from Agilent (Santa Clara, CA, USA)

### 2.3. Procedures

#### 2.3.1. Modification of the Electrode Surface with Metal Dioxide NPs Dispersion

Prior to the modification, GCE surface was polished on alumina slurry (particle size of 0.05 µm) and then rinsed thoroughly with acetone and distilled water. Drop-casting of 5 μL of SnO_2_–CeO_2_ NPs dispersion and further air-drying for 10 min were used for the GCE surface modification.

#### 2.3.2. Voltammetric and Chronoamperometric Measurements

Voltammetry was performed in Britton–Robinson buffer (BRB) of pH 2.0–12.0. Five voltammograms of supporting electrolyte were recorded prior to the analyte addition. The total volume of solution in the electrochemical cell was 4.0 mL. Cyclic voltammograms were registered from 0.0 to 1.3 V with a potential scan rate of 0.10 V s^−1^.

Differential pulse voltammograms were recorded from 0.2 to 1.1 V with a potential step of 5 mV and a scan rate of 0.010 V s^−1^. The pulse parameters were preliminary optimized. Baseline correction using NOVA 1.7.8 software (Eco Chemie B.V., Utrecht, The Netherlands) was applied for data presentation.

Chronoamperometry was used for the evaluation of the GCE electroactive surface area based on the electrooxidation of 1.0 mM hexacyanoferrate(II) ions in 0.1 M KCl. Electrolysis was performed at 450 mV for 75 s.

#### 2.3.3. Electrochemical Impedance Spectroscopy

Hexacyanoferrate(II)/(III) ions mixture (1.0 mM in 0.1 M KCl) was used as a redox probe. Impedance spectra were recorded at a polarization potential of 230 mV (calculated as a half-sum of the redox probe redox potentials) in the frequency range of 10,000–0.04 Hz at an applied sine potential amplitude of 5 mV. The Nyquist plots were fitted using Randles’ equivalent circuit consisting of the electrolyte (*R*_s_) and electron transfer resistance (*R*_et_), constant phase element (*Q*), and Warburg impedance (*W*) [59]. The χ^2^ parameter was used for the characterization of fitting error.

#### 2.3.4. Soft Drinks Analysis

Commercial soft drinks were used as real samples. Filtration using Captiva Econofilter with a nylon membrane of 0.45 µm pore size was applied prior to analysis. An aliquot portion of the sample (30–70 µL) was inserted in the electrochemical cell containing BRB pH 2.0 (3970–3930 µL), and the differential pulse voltammogram was recorded from 0.2 to 1.2 V at a pulse amplitude of 100 mV and a pulse time of 25 ms with a potential step of 5 mV and a scan rate of 0.010 V s^−1^.

#### 2.3.5. Statistical Treatment of the Data

All electrochemical and scanning electron microscopy measurements were performed in five replications (three replications in HPLC). The statistical treatment of the results was conducted at 95% probability. All data were shown as an average value ± coverage interval. Random errors were evaluated based on the relative standard deviation values. Dependent *t*-test for paired samples and *F*-test were used for the sensor validation.

Linear regression and statistical analysis were carried out using OriginPro 8.1 software (OriginLab, Northampton, MA, USA).

## 3. Results and Discussion

### 3.1. Voltammetric Behavior of SY FCF on Bare and MO_2_-NPs-Modified GCE

**Figure 2:**
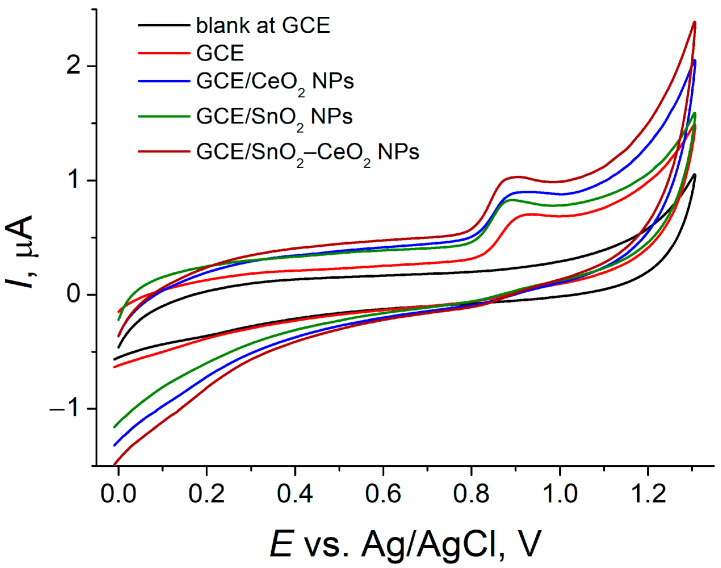
Cyclic voltammograms of 10 μM SY FCF at the bare GCE and GCE modified with water dispersions of MO_2_ NPs. Blank is BRB pH 2.0. υ = 0.10 V s^−1^.

Cyclic voltammetry in BRB pH 2.0 has shown that SY FCF is electroactive on bare and modified electrodes. An irreversible oxidation peak at 906 mV (Figure 2) has been observed at the bare GCE. Modification of the electrode surface with water dispersions of MO_2_ NPs provides a cathodic shift of the oxidation potential on ≈30 mV and a 1.2–1.6-fold increase in the oxidation peak currents (Figure 2). The mixture of SnO_2_ and CeO_2_ NPs has shown a more pronounced effect compared to individual NPs. Nevertheless, these changes are insufficient from an analytical point of view.

Therefore, the surfactants of various natures have been tested as dispersive media for the SnO_2_ and CeO_2_ NPs mixture. In this case, there are two aspects of surfactant action to be considered. The first one is the stabilization of NP dispersion and prevention of their aggregation, which provides a smaller size of NP, as shown in a further scanning electron microscopy study. The second point is the possibility of a hydrophobic or electrostatic interaction of the surfactant at the electrode surface with the analyte, leading to its preconcentration.

Cationic HDPB, anionic SLS, and non-ionic Brij^®^ 35 have been tested as dispersive media. Based on previous experience [24], 0.10 mM water solutions of surfactants have been used. SLS on contrary to HDPB and Brij^®^ 35 media does not allow us to obtain stable dispersion.

The application of SnO_2_ and CeO_2_ NPs mixture dispersed in HDPB and Brij^®^ 35 as an electrode surface modifier leads to changes in the SY FCF voltammetric response. GCE/SnO_2_–CeO_2_ NPs–Brij^®^ 35 gives a well-shaped oxidation peak at 876 mV with a current of 0.42 ± 0.02 μA, that is, a 2.3-fold increased vs. bare GCE (0.18 ± 0.03 μA) and 1.5-fold higher than GCE/SnO_2_–CeO_2_ NPs (0.285 ± 0.005 μA). GCE/SnO_2_–CeO_2_ NPs–HDPB has shown an insignificant anodic shift of the SY FCF oxidation peak potential to 927 mV and a 5.8-fold increase in the oxidation peak current compared to bare GCE. Such a significant effect can be explained by the interaction of HDPB with SY FCF via the electrostatic attraction between positively charged heads of HDPB and negatively charged fragments at pH 2.0 SY FCF due to the dissociation of sulfonate groups [60].

The effect of HDPB as an electrode surface co-modifier on the voltammetric response of SY FCF has been studied (Table 2).

**Figure 3:**
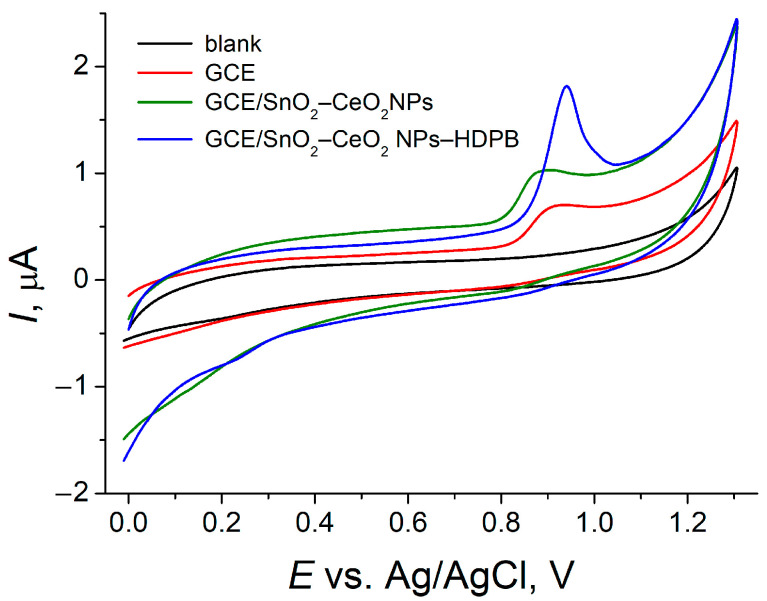
Cyclic voltammograms of 10 μM SY FCF at the bare GCE and MO_2_-NPs-modified GCE in BRB pH 2.0. υ = 0.10 V s^−1^.

As one can see, GCE/HDPB gives a 30 mV anodic shift of the SY FCF oxidation peak and a 3.6-fold increase in the oxidation currents. Data obtained at the GCE modified with SnO_2_ NPs or CeO_2_ NPs dispersed in HDPB indicate the synergetic effect of metal oxide NPs and HDPB. GCE/SnO_2_–CeO_2_ NPs–HDPB shows the highest oxidation peak currents and the best shape of the SY FCF voltammograms (Figure 3), which is caused by an increase in the electroactive surface area of the electrode and the electron transfer rate vs. bare GCE (see Section 3.2.2), as well as by electrostatic interactions between HDPB and SY FCF.

### 3.2. Characterization of the Electrodes

#### 3.2.1. Field Emission Scanning Electron Microscopy

**Figure 4:**
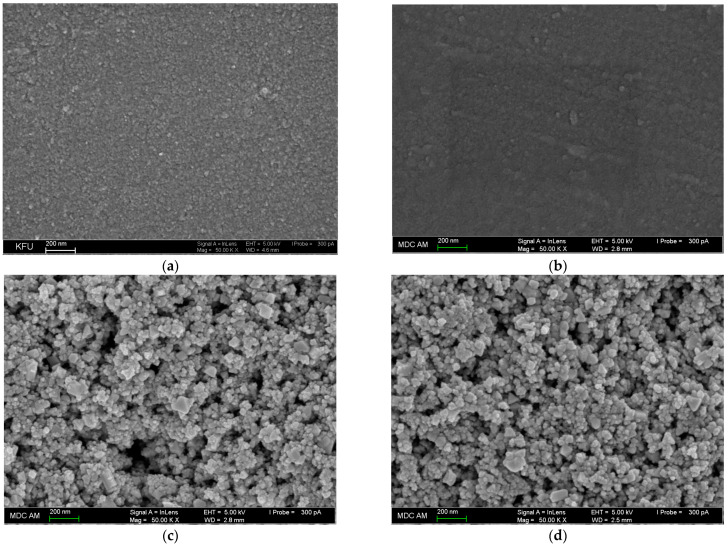
Field emission scanning electron microscopy images of the electrode surface: (**a**) bare GCE; (**b**) GCE/HDPB; (**c**) GCE/SnO_2_–CeO_2_ NPs; (**d**) GCE/SnO_2_–CeO_2_ NPs–HDPB. Magnification is 50,000×.

The electrodes’ surface morphology has been studied via field emission scanning electron microscopy (Figure 4). Bare GCE shows a typical unstructured surface of low roughness (Figure 4a). HDPB forms thin-film coverage (Figure 4b). SnO_2_–CeO_2_ NPs mixture dispersed in water is represented by spherical particles of 20–45 nm in combination with pyramidal structures up to 125 × 165 nm size (Figure 4c). In the case of SnO_2_–CeO_2_ NPs dispersion in HDTP, the spherical NPs diameter is decreased to 12–40 nm, with a prevalence of smaller NPs of 12–20 nm and single inclusions of aggregates up to 150 nm (Figure 4d). The use of HDPB as a dispersive agent leads to a smaller size of the metal oxides NPs, as has also been confirmed on dispersions of SnO_2_ or CeO_2_ NPs in water and HDPB (Figure S1). These data agree well with the literature data [27,61,62]. MO_2_-NPs-modified electrodes demonstrate porous coverage with channels, which indicates a high surface area.

#### 3.2.2. Evaluation of the Electroactive Surface Area and Electron Transfer Properties

**Figure 5:**
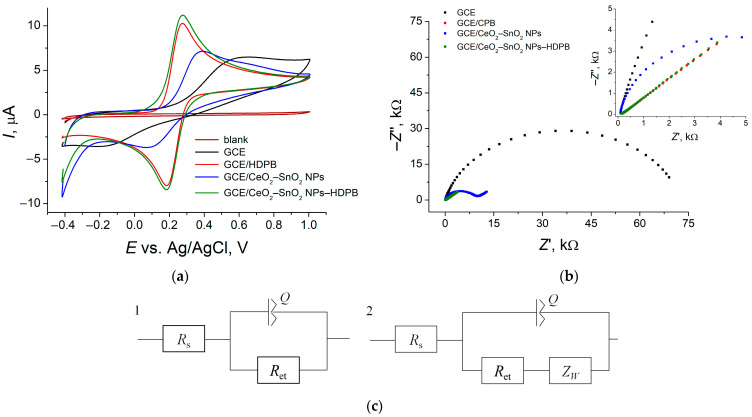
(**a**) Cyclic voltammograms of 1.0 mM hexacyanoferrate(II) ions at the various electrodes. Supporting electrolyte is 0.1 M KCl, υ = 0.10 V s^−1^. (**b**) Nyquist plot for the various electrodes. Redox probe is 1.0 mM hexacyanoferrate(II)/(III) ions; supporting electrolyte is 0.1 M KCl, *E* = 230 V; frequency range from 10,000 to 0.04 Hz; amplitude is 5 mV. (**c**) Randles equivalent circuits used for the impedance spectra fitting for the bare GCE (1) and modified electrodes (2). *R*_s_—active electrolyte resistance; *R*_et_—electron transfer resistance; *Q*—constant phase element; and *W*—Warburg impedance.

The electroactive surface area of the electrodes has been evaluated using hexacyanoferrate(II) ions oxidation in 0.1 M KCl. Chronoamperometry and the Cottrell equation [63] have been applied in the case of bare GCE (Figure S2) due to the absence of full reversibility of the electrochemical reaction (Figure 5a). The improvement of electrooxidation reversibility has been observed for the GCE/SnO_2_–CeO_2_ NPs, as cathodic-to-anodic peak potential separation and redox currents ratios indicate (Figure 5a). The ideally reversible electrode reaction has been registered at the HDPB-modified electrodes caused by the electrostatic attraction between positively charged HDPB and negatively charged hexacyanoferrate(II) ions. Thus, cyclic voltammetry data and the Randles–Ševčík equation [63] have been used for the calculation of the electroactive surface area. The quantitative data are summarized in Table 3.

Electron transfer properties have been characterized via electrochemical impedance spectroscopy using a hexacyanoferrate(II)/(III) ions mixture as a redox probe. Typical Nyquist plots are presented in Figure 5b. As one can see, the semicircle diameter at high frequencies is dramatically decreased for the modified electrodes, reaching the minimal values for the electrodes covered with HDPB and SnO_2_–CeO_2_ NPs–HDPB. These results indicate a significant decrease in the electron transfer resistance, which is explained by electrostatic interactions between HDPB at the electrode surface with the redox probe, similar to that reported in [26,27,64]. Impedance spectra fitting results obtained using Randles’ equivalent circuit (Figure 5c) are presented in Table 3.

The heterogeneous electron transfer rate constants (*k*_et_) (Table 3) have been calculated using electrochemical impedance data and Equation (1) [65]:

(1) $$k_{\mathrm{et}}=\frac{RT}{F^{2}n^{2}R_{\mathrm{et}}Ac}$$

A 38-fold increase in the heterogeneous rate constants for the GCE/SnO_2_–CeO_2_ NPs–HDPB compared to bare GCE confirms the effectivity of the developed electrode in the electron transfer.

### 3.3. Electrooxidation of SY FCF at the GCE/SnO_2_–CeO_2_ NPs–HDPB

#### 3.3.1. Effect of the Supporting Electrolyte pH

**Figure 6:**
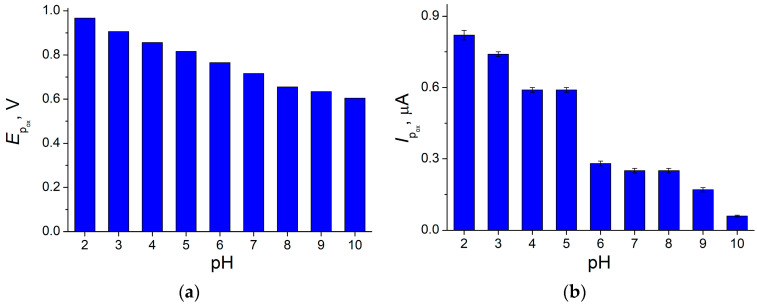
The changes of the voltammetric characteristics of 10 μM SY FCF at the GCE/SnO_2_–CeO_2_ NPs–HDPB in the BRB of various pH: (**a**) effect on the oxidation peak potential; (**b**) effect on the oxidation peak currents. Data obtained by cyclic voltammetry at υ = 0.10 V s^−1^.

The effect of BRB pH on the voltammetric parameters of SY FCF has been studied. The dye is stable in a wide range of pH, especially in acidic and neutral media [66]. The oxidation potential is gradually decreased as pH increases, indicating participation of the protons in the electrode reaction (Figure 6a). The absence of cathodic steps in the whole pH range studied confirms the irreversibility of the SY FCF electrooxidation. The slope of the plot *E* vs. pH in acidic and neutral media is 36 mV; i.e., the number of electrons participating in the electrode reaction is twice that of the number of protons, which is in line with the reported data for the carbon-paste electrode modified by silica impregnated with cetylpyridinium chloride [67], GCE modified with water-compatible molecularly imprinted ionic liquid polymer–ionic liquid functionalized graphene composite [68], and GCE modified with nanocomposite of nickel and electrochemically reduced graphene oxide [69]. The oxidation peak is fully disappeared at pH 11.0, which is probably caused by dye ionization with the phenolate ion formation and its oxidation by air oxygen.

The oxidation peak currents are slowly decreased with the pH increase (Figure 6b), which agrees well with the published data [67]. In acidic medium, the ion pairs are formed between the SY FCF existing as the dianion and positively charged HDPB. The other experiments have been carried out at pH 2.0.

#### 3.3.2. Effect of the Potential Scan Rate

**Figure 7:**
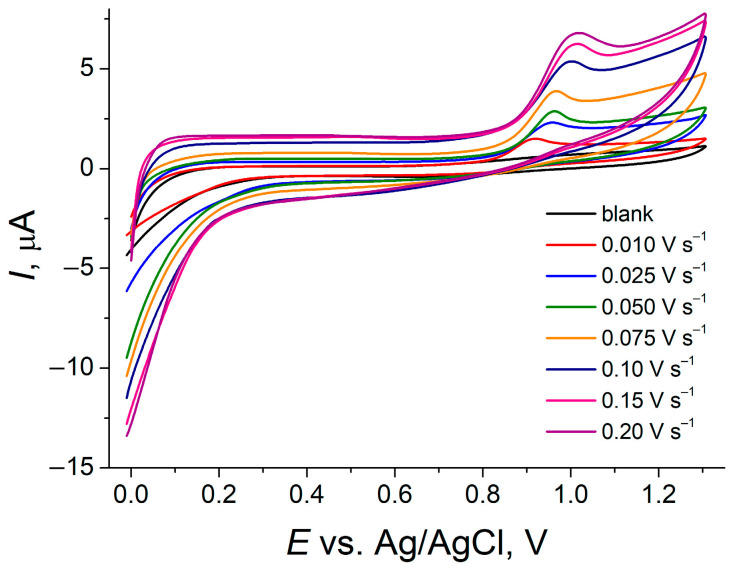
Cyclic voltammograms of 50 μM SY FCF at the GCE/SnO_2_–CeO_2_ NPs–HDPB in BRB pH 2.0 at various potential scan rates.

Effect of the potential scan rate on the voltammetric parameters of SY FCF has been studied in BRB pH 2.0. Oxidation proceeds irreversibly, independently of the potential scan rate (Figure 7).

The oxidation peak potential is anodically shifted with the increase in potential scan rate (Equation (2)):

*E* [V] = (1.069 ± 0.009) + (0.040 ± 0.003)lnυ [V s^−1^]    *R*^2^ = 0.9663. (2)

The linear plot of the oxidation peak currents vs. the square root from potential scan rate (Equation (3)), as well as the slope of ln*I* vs. lnυ equal to 0.41 (Equation (4)), indicate the diffusion-controlled electrooxidation of SY FCF at the GCE/SnO_2_–CeO_2_ NPs–HDPB.

*I* [µA] = (0.31 ± 0.03) + (0.154 ± 0.003)υ^½^ [mV s^−1^]   *R*^2^ = 0.9974, (3)

ln*I* [µA] = (1.57 ± 0.03) + (0.41 ± 0.01)lnυ [V s^−1^]   *R*^2^ = 0.9964. (4)

The oxidation parameters have been calculated as the basis of voltammetric data treatment. The anodic transfer coefficient of 0.60 has been found in the Tafel plot at a low potential scan rate (0.010 V s^−1^). Then, the number of electrons was calculated using Equation (5) valid for the irreversible diffusion-driven electrochemical process [63]:

*E*_p_ − *E*_p/2_ [mV] = 47.7/α_a_*n*. (5)

**Scheme 1:**
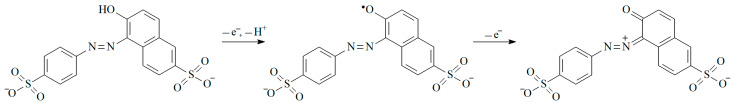
Scheme of the SY FCF electrooxidation at the GCE/SnO_2_–CeO_2_ NPs–HDPB.

Thus, the electrooxidation of SY FCF at the GCE/SnO_2_–CeO_2_ NPs–HDPB involves two electrons. Taking into account what was mentioned above regarding the electron-to-proton ratio, two electron and one proton transfer occurs. Similar data have been reported earlier for another modified electrode [67,69,70,71]. The following reaction scheme has been suggested (Scheme 1).

The diffusion coefficient and the standard heterogeneous electron transfer rate constant of SY FCF have been calculated as (2.3 ± 0.1) × 10^−6^ cm^2^ s^−1^ and (8.1 ± 0.3) × 10^−4^ cm s^−1^ using well-known equations (Equations (S1) and (S2), respectively) for the diffusion-controlled irreversible electrooxidation [63,72]. These parameters are significantly higher than those obtained for the GCE modified with multi-walled carbon nanotubes and poly(4-aminobenzoic acid) [73].

### 3.4. Analytical Performance of the Developed Voltammetric Sensor

#### 3.4.1. Optimization of Differential Pulse Voltammetry Conditions

Taking into account the peculiarities of the SY FCF electrooxidation, differential pulse voltammetry has been used for analytical purposes. First of all, the optimization of pulse parameters has been carried out. The oxidation peak potential is insignificantly shifted to lower values with the increase in the pulse amplitude and pulse time (Figure S3a), and it achieves its minimal value at an amplitude of 100 mV and time of 100 ms. SY FCF oxidation peak currents are increased with the growth of pulse amplitude and slowly decreased with the increase in the pulse time (Figure S3b). The best response of SY FCF has been observed at a pulse amplitude of 100 mV and a time of 25 ms.

The electrochemical window, especially the start potential, affects the target analyte response in voltammetry. Varying the start potential in the range of 0.0–0.4 V, the optimal value has been found as 0.2 V. Therefore, the electrochemical window from 0.2 to 1.2 V has been used.

#### 3.4.2. Analytical Characteristics of SY FCF

**Figure 8:**
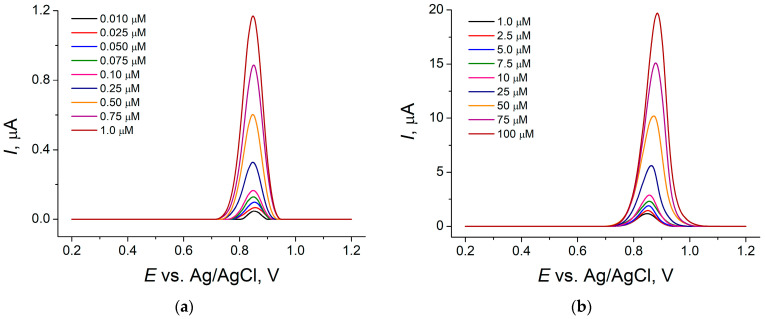
Baseline-corrected differential pulse voltammograms of SY FCF at the GCE/SnO_2_–CeO_2_ NPs–HDPB in BRB pH 2.0: (**a**) concentration range of 0.010–1.0 μM; (**b**) concentration range of 1.0–100 μM. Pulse amplitude is 100 mV, pulse time is 25 ms, potential scan rate is 0.010 V s^−1^.

Figure 8 represents differential pulse voltammograms of SY FCF of various concentrations at the GCE/SnO_2_–CeO_2_ NPs–HDPB in BRB pH 2.0. The oxidation peak at 850 mV is linearly changed with the increase in the dye concentration. Two linear dynamic ranges, from 0.010 to 1.0 μM and from 1.0 to 100 μM SY FCF, have been obtained and are described with the Equations (6) and (7), respectively.

*I* [µA] = (0.041 ± 0.003) + (113.1 ± 0.7) × 10^4^ *c* [M]   *R*^2^ = 0.9997, (6)

*I* [µA] = (0.94 ± 0.04) + (188.1 ± 0.9) × 10^3^ *c* [M]   *R*^2^ = 0.9998. (7)

The sensitivity of the sensor response is 113.1 ± 0.7 and 188.1 ± 0.9 µA M^−1^ for the first and the second analytical ranges, respectively, which is higher than that reported for the electrodes modified with hierarchical flower-like NiCo_2_O_4_ nanoplates [51], muti-walled carbon nanotube/poly(4-aminobenzoic acid) [73], poly(Alizarin Red-S)/functionalized multi-walled carbon nanotubes [74], flower-like MoS_2_ [75], etc. The detection limit of SY FCF calculated as 3SD_a_/*b* equals 8.0 nM.

The achieved analytical characteristics are among the best ones presented for metal oxide nanomaterials-based electrodes (Table 1) and other modified electrodes (Table 4). Moreover, the developed method does not require a preconcentration step, which significantly reduces measurement time (adsorptive concentration usually takes about 3–5 min) and excludes the possibility of the co-adsorption of other components from the real samples. Another advantage is the simplicity of the electrode surface modifier’s preparation and immobilization.

The sensor accuracy has been tested using model SY FCF solutions on five levels of concentration covering the whole analytical range (Table 5). The relative standard deviation of 0.51–5.5% indicates the absence of random errors of SY FCF quantification. Furthermore, these data confirm the high reproducibility of the sensor response due to surface renewal after each measurement. Recovery of 100% proves the high accuracy of the developed sensor.

The selectivity of the GCE/SnO_2_–CeO_2_ NPs–HDPB response to SY FCF has been evaluated at a 0.50 μM level. Typical components of the real samples, such as inorganic ions and saccharides, as well as antioxidants, preservatives, flavoring, and other dyes, were tested as potential interferences. Several of them (ascorbic acid, vanillin, carminic acid, tartrazine, and brilliant blue FCF) are electroactive at the GCE/SnO_2_–CeO_2_ NPs–HDPB (Figure S4); others are silent on the voltammograms. Ascorbic and carminic acids are oxidized at less-positive potentials and two well-separated peaks are obtained in the case of their mixtures with SY FCF (Figure S4a and S4c, respectively). Vanillin oxidation peak potential is almost similar to SY FCF. Nevertheless, the sensitivity of the GCE/SnO_2_–CeO_2_ NPs–HDPB response to vanillin is significantly lower than for the SY FCF. The oxidation peak of vanillin is fully disappeared at 0.10 μM (Figure S4b). Tartrazine and brilliant blue FCF are oxidized at more-positive potentials than SY FCF (Figure S4d and S4e, respectively), and peak potential separation is more than 100 mV. Unfortunately, the full resolution of dyes’ oxidation peaks cannot be achieved, leading to the redistribution of the oxidation currents of each dye in the mixture. Happily, similar to vanillin, the oxidation currents of tartrazine and brilliant blue FCF are significantly lower than those of the SY FCF. Therefore, the interference effect is completely removed in the presence of 0.050 and 0.10 μM of tartrazine and brilliant blue FCF, respectively (Figure S4d and S4e, respectively). The corresponding oxidation peak potentials and tolerance limits are summarized in Table 6. Thus, a simple dilution of real sample can eliminate the potential interference effect from other electroactive compounds.

#### 3.4.3. Soft Drinks Analysis

**Figure 9:**
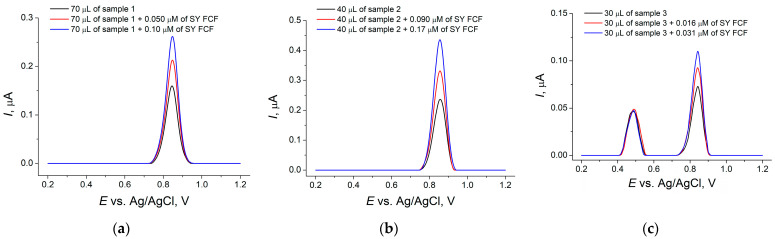
Baseline-corrected differential pulse voltammograms of soft drinks at the GCE/SnO_2_–CeO_2_ NPs–HDPB in BRB pH 2.0: (**a**) sample 1 with SY FCF additions; (**b**) sample 2 with SY FCF additions; (**c**) sample 3 with SY FCF additions. Pulse amplitude is 100 mV, pulse time is 25 ms, potential scan rate is 0.010 V s^−1^.

The sensor developed has been successfully applied in the soft drinks analysis. All samples demonstrate a well-defined oxidation peak at 850 mV (Figure 9) corresponding to SY FCF, as the standard addition method confirms (Table S1). The recovery values of 99.8–101% clearly indicate the absence of matrix effects and the linearity of the sensor response towards SY FCF in the presence of the soft drinks. Sample 3 (Figure 9c) also shows an oxidation peak at 485 mV that does not affect SY FCF determination.

Table 7 represents the results of the voltammetric quantification of SY FCF in soft drinks and their validation with the standard HPLC method [87]. The data obtained with the two methods are almost the same, which confirms the practical applicability of the sensor developed in real practice. The one-sample Student’s *t*-test data are lower than the critical value (2.45), indicating the absence of systematic errors of determination. The *F*-test results do not exceed critical values of 6.94 (for samples 1 and 2) and 19.25 (for sample 3), which implies the similar precision of both methods.

## 4. Conclusions

A combination of tin and cerium dioxide NPs dispersed in cationic surfactant HDPB has been shown, for the first time, to be an effective sensing layer of the voltammetric sensor for SY FCF. The electrochemical characteristics of the sensor (electroactive surface area and electron transfer rate constant) allow us to conclude that it can be also applied to other analytes, which emphasizes the practical applicability of electrochemical sensors based on semiconducting transition metal oxide NPs. Further developments in the field could be focused on the fabrication of screen-printed electrodes with this type of coverage, which would significantly simplify the analytical procedure and increase the throughput of the system. The absence of an adsorptive preconcentration minimizes the risk of other components’ co-adsorption and the associated interference effects, also reducing measurement time.

## Figures and Tables

**Table 1:** Figures of merit of electrochemical sensors based on metal oxide nanomaterials for SY FCF quantification.

Sensor	Method	pH	Detection Limit, µM	Linear Dynamic Range, µM	Ref.
Nd_2_O_3_–CPE ^1^	AdASWV ^2^	3.0	0.090	0.20–2.0	[46]
MoO_2_–CPE	AdASWV	4.1	0.060	0.16–2.0	[47]
ZnO nanoflowers–CPE	AdASWV	5.0	2.21 × 10^−4^	0.0011–0.022, 0.022–0.155	[48]
La_2_O_3_–TiO_2_/CPE	AdASWV	2.0	0.02	0.26–4.5	[49]
Fe-Zr oxides/GCE ^3^	DPV ^4^	2.0	5.7	19–270	[50]
Hierarchical flower-like NiCo_2_O_4_ nanoplates/SPGE ^5^	DPV	7.0	0.008	0.02–145	[51]
MnO_2_ nanorods–ERGO ^6^/GCE	AdASDLSV ^7^	4.5	0.002	0.01–2.0, 2.0–10, 10–100	[52]
Dy_2_O_3_ NPs–MWCNTs(ox) ^8^/GCE	SWV ^9^	7.0	3.5 × 10^−4^	0.0010–0.14	[53]
Pr_6_O_11_@Dy_2_O_3_−Carbon nanofibers/GCE	SWV	7.0	3.12 × 10^−4^	0.0010–0.035	[54]
Mesoporous TiO_2_ NPs–Graphene–CPE	AdASWV	1.0	0.006	0.020–2.05	[55]
Fe_3_O_4_@SiO_2_ NPs–MWCNTs-COOH ^10^–CPE	AdSSWV ^11^	6.0	0.050	0.50–100	[56]
Cu@Cu_2_O-B and N codoped porous carbon/GCE	DPV	6.5	0.0024	0.01–8	[57]
Electrodeposited ZnO/Cysteinic acid/GCE	AdADPV ^12^	5.0	0.031	0.10–3.0	[58]

^1^ Carbon paste electrode. ^2^ Adsorptive anodic square-wave voltammetry. ^3^ Glassy carbon electrode. ^4^ Differential pulse voltammetry. ^5^ Screen-printed graphite electrode. ^6^ Electrochemically reduced graphene oxide. ^7^ Adsorptive anodic second-order derivative linear sweep voltammetry. ^8^ Multi-walled carbon nanotubes after acid treatment. ^9^ Square-wave voltammetry. ^10^ Carboxylated multi-walled carbon nanotubes. ^11^ Adsorptive stripping square-wave voltammetry. ^12^ Adsorptive anodic differential pulse voltammetry.

**Table 2:** Voltammetric characteristics of 10 μM SY FCF on bare and modified GCE (*n* = 5; *p* = 0.95).

Electrode	*E*_pox_, mV	*I*_pox_, μA
Bare GCE	906	0.18 ± 0.03
GCE/SnO_2_–CeO_2_ NPs	876	0.285 ± 0.005
GCE/SnO_2_–CeO_2_ NPs–HDPB	927	1.05 ± 0.06
GCE/HDPB	936	0.45 ± 0.01
GCE/SnO_2_ NPs–HDPB	927	0.67 ± 0.04
GCE/CeO_2_ NPs–HDPB	937	0.56 ± 0.05

**Table 3:** Electroactive surface area and electrochemical impedance parameters of the electrodes under consideration (*n* = 5; *p* = 0.95).

Electrode	*A*, mm^2^	*R*_s_, Ω	*R*_et_, kΩ	*Q*, µΩ^−1^	*n*	*W*, µΩ^−1^	χ^2^	*k*_et_, cm s^−1^
Bare GCE	8.9 ± 0.3	245 ± 5	72 ± 3	3.7 ± 0.2	0.789	-	0.03	4.15 × 10^−5^
GCE/HDPB	32 ± 1	35 ± 1	0.19 ± 0.03	96 ± 4	0.365	290 ± 7	0.01	4.38 × 10^−3^
GCE/SnO_2_–CeO_2_ NPs	36 ± 2	84 ± 2	9.0 ± 0.7	827 ± 9	0.887	264 ± 10	0.05	8.2 × 10^−5^
GCE/SnO_2_–CeO_2_ NPs–HDPB	40 ± 2	106 ± 3	0.42 ± 0.05	243 ± 2	0.466	138 ± 1	0.006	1.58 × 10^−3^

**Table 4:** Analytical characteristics of SY FCF on various electrochemical sensors.

Sensor	Method	Detection Limit, µM	Linear Dynamic Range, µM	Ref.
Poly(L-methionine)/Reduced graphene oxide/SPCE ^1^	AdADPV	0.048	1–10 and 10–50	[70]
Exfoliate graphite/GCE	Amperometry	0.0085	0.028–30	[71]
Poly(4-aminobenzoic acid)/MWCNTs ^2^/GCE	DPV	0.0023	0.010–0.75 and 0.75–5.0	[73]
Functionalized MWCNTs/poly(Alizarin Red-S)/GCE	SWV	0.16	0.50–256	[74]
Molecularly imprinted polyglycine/Pencil graphite electrode	SWV	0.753	2–100	[75]
Flower-like MoS_2_/SPE ^3^	DPV	0.04	0.16–2.0	[76]
3D reduced graphene oxide loaded with cauliflower-like MoS_2_/GCE	DPV	0.0176	0.05–10 and 10–60	[77]
3D raspberry-like In^3+^/NiO hierarchical nanostructures/GCE	DPV	0.0027	0.01–700	[78]
CuNiFe_2_O_4_ nanospheres–carbon paste electrode	DPV	0.15	0.5–150	[79]
Au NPs/PDDA ^4^–graphene/GCE	DPV	0.002	0.006–5.0	[80]
Ag NPs-Co_3_O_4_/Functionalized MWCNTs/SPCE	DPV	0.0008	0.06–60	[81]
Molecularly imprinted polymer-Au NPs/Indium-tin oxide	SWV	0.0016	0.010–100	[82]
Graphene oxide nanosheets–carbon paste electrode	DPV	0.010	1–20	[83]
Electrodeposited grapene oxide/Pencil graphite electrode	DPV	0.057	0.5–10	[84]
Fine-grained graphite powder/Carbon paper electrode	AdADPV	0.00078	0.005–1.0	[85]
Laccase/Poly(acrylamide-co-ethyl methacrylate) membrane/GCE	AdADPV	0.02	0.08–10	[86]

^1^ Screen printed carbon electrode. ^2^ Multi-walled carbon nanotubes. ^3^ Screen-printed electrode. ^4^ Poly(diallyldimethylammonium chloride).

**Table 5:** SY FCF quantification using GCE/SnO_2_–CeO_2_ NPs–HDPB in BRB pH 2.0 (*n* = 5; *p* = 0.95).

Added, μM	Found, μM	RSD, %	*R*, %
0.010	0.0100 ± 0.0007	5.5	100 ± 7
0.25	0.249 ± 0.006	1.9	100 ± 2
1.0	1.00 ± 0.01	1.0	100 ± 1
7.5	7.51 ± 0.09	0.97	100 ± 1
100	100 ± 1	0.51	100 ± 1

**Table 6:** Oxidation peak potentials and tolerance limits of the interferences for the determination of 0.50 μM SY FCF at the GCE/SnO_2_–CeO_2_ NPs–HDPB in BRB pH 2.0 (*n* = 5; *p* = 0.95).

Interference	*E*_pox_, mV	Tolerance Limit, μM
K^+^	―	500
Mg^2+^	―	500
Ca^2+^	―	500
NO_3_^−^	―	500
Cl^−^	―	500
SO_4_^2−^	―	500
Glucose	―	50
Rhamnose	―	50
Sucrose	―	50
Ascorbic acid	391	25
Sorbic acid	―	50
Vanillin	870	0.10
Carminic acid	557	2.5
Riboflavin	―	50
Tartrazine	996	0.050
Brilliant blue FCF	965	0.10

**Table 7:** SY FCF contents in soft drinks (*p* = 0.95).

Sample	SY FCF Contents, mg L^−1^				*t*-Test	*F*-Test
	Voltammetry	RSD, %	HPLC	RSD, %		
1	2.6 ± 0.1	4.2	2.6 ± 0.3	5.5	0.341	1.63
2	8.0 ± 0.1	1.2	8.1 ± 0.6	2.7	1.18	4.98
3	1.87 ± 0.08	3.5	1.86 ± 0.06	1.4	0.748	6.82

## Data Availability

The data presented in this study are available in the electronic Supplementary Materials.

## Supplementary Materials

The following supporting information can be downloaded at https://www.mdpi.com/article/10.3390/s24030930/s1. Figure S1: Field emission scanning electron microscopy images of the electrode surface: (a) GCE//SnO_2_ NPs; (b) GCE/SnO_2_ NPs–HDPB; (c) GCE/CeO_2_ NPs; (d) GCE/CeO_2_ NPs–HDPB. Magnification is 50,000×; Figure S2: Chronoamperograms of hexacyanoferrate(II) ions in 0.1 M KCl on the bare GCE at 450 mV. The inset is the plot of *I* vs. *t*^−1/2^; Equations (S1) and (S2): Equations used for the calculation of SY FCF diffusion coefficient and standard heterogeneous electron transfer rate constant; Figure S3: Effect of pulse parameters on the voltammetric characteristics of 1.0 µM SY FCF at the GCE/SnO_2_–CeO_2_ NPs–HDPB in BRB pH 2.0: (a) the changes in the oxidation peak potential; (b) the changes in the oxidation peak currents. υ = 0.010 V s^−1^; Figure S4: Baseline-corrected differential pulse voltammograms of interferences and their mixtures with SY FCF at the GCE/SnO_2_–CeO_2_ NPs–HDPB in BRB pH 2.0: (a) ascorbic acid; (b) vanillin; (c) carmnic acid; (d) tartrazine; (e) brilliant blue FCF. Pulse amplitude is 100 mV, pulse time is 25 ms, potential scan rate is 0.010 V s^−1^; Table S1: Recovery of SY FCF in soft drinks (*n* = 5; *p* = 0.95).
