# Supplementary material for: Voltammetric Sensor Based on the Combination of Tin and Cerium Dioxide Nanoparticles with Surfactants for Quantification of Sunset Yellow FCF
Source: Sensors (Basel). 2024 Jan 31;24(3):930. doi: 10.3390/s24030930 (PMC10857103; doi:10.3390/s24030930)
Supplement: Supplementary file 1 [file sensors-24-00930-s001.zip › sensors-2848047-supplementary.pdf]

Supplementary Materials

Voltammetric Sensor Based on the Combination of Tin and Cerium Dioxide Nanoparticles with Surfactants for Quantification of Sunset Yellow FCF

Liliya Gimadutdinova, Guzel Ziyatdinova,* and Rustam Davletshin

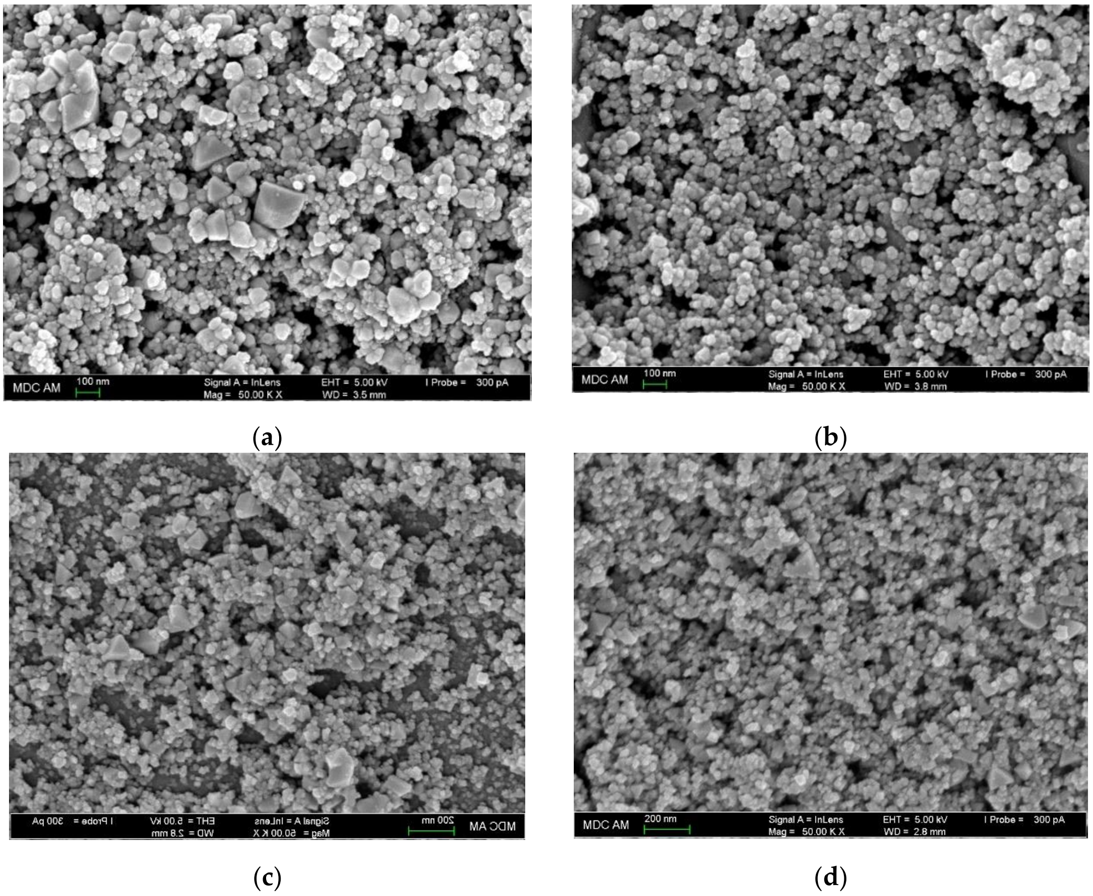

Figure S1. Field emission scanning electron microscopy images of the electrode surface: (**a**) GCE//SnO_2 NPs; (**b**) GCE/SnO_2 NPs–HDPB; (**c**) GCE/CeO_2 NPs; (**d**) GCE/CeO_2 NPs–HDPB. Magnification is 50,000×.

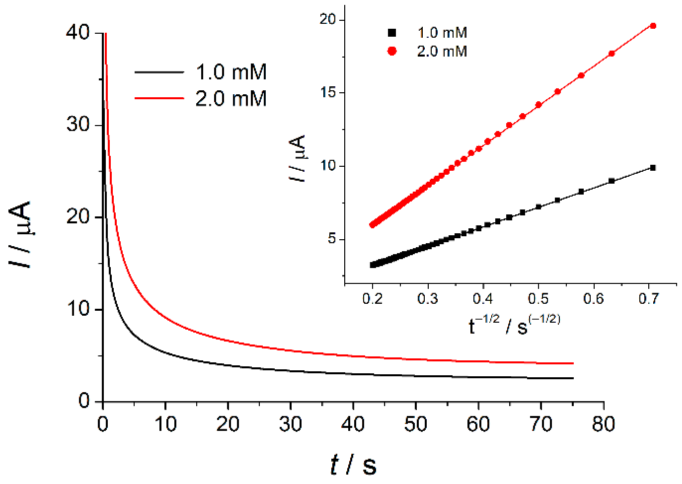

Figure S2. Chronoamperograms of hexacyanoferrate(II) ions in 0.1 M KCl on the bare GCE at 450 mV. The inset is the plot of I vs. $t^{-1/2}$.

Diffusion coefficien has been calculated from the Equation S1

$$I_{\mathrm{p_{ox}}} = \pi^{1/2}\chi(bt)nFAcD^{1/2}\left(\frac{\alpha_{\mathrm{a}} n_{\alpha} F}{RT}\right)^{1/2}\upsilon^{1/2} \qquad \text{(S1)}$$

where I_{ox} – oxidation peak current (A), $\chi(bt)$ – normalized current for sweep experiments with an irreversible system, n – the number of electrons participating in oxidation, F – the Faraday constant (C mol^{-1}), A – the electrode surface area (cm^2), c – concentration (mol cm^{-3}), D – diffusion coefficient (cm^2 s^{-1}), α_a – anodic transfer coefficient, n_α – the number of electrons involved in the rate-determining step, R – the gas constant (J mol^{-1} K^{-1}), T – temperature (K) and υ – potential scan rate (V s^{-1}).

The standard heterogeneous electron transfer rate constant k^0 has been calculated using Equation S2

$$k^0 = 2.415e^{-\frac{0.02F}{RT}} D^{1/2}(E_{\mathrm{p}} - E_{\mathrm{p}_{1/2}})^{-1/2}\upsilon^{1/2} \qquad \text{(S2)}$$

where k^0 is the standard heterogeneous electron transfer rate constant (cm s^{-1}), D – diffusion coefficient (cm^2 s^{-1}), E – the oxidation potential (V), $E_{1/2}$ – the oxidation half-wave potential (V), other symbols have the same meaning as in Equation S2.

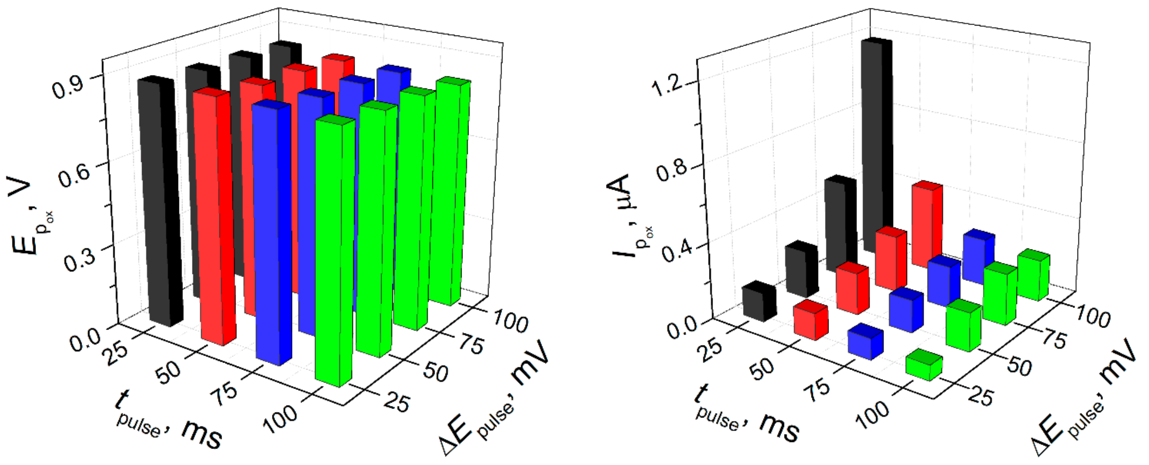

(**a**) (**b**)

Figure S3. Effect of pulse parameters on the voltammetric characteristics of 1.0 μM SY FCF at the GCE/SnO_2–CeO_2 NPs–HDPB in BRB pH 2.0: (**a**) the changes in the oxidation peak potential; (**b**) the changes in the oxidation peak currents. υ = 0.010 V s^{-1}.

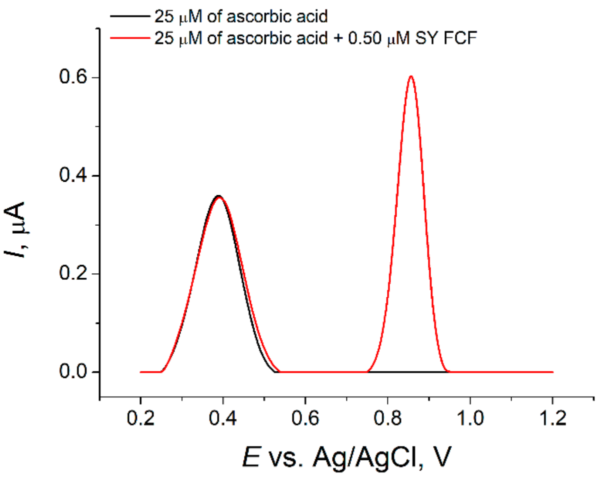

(**a**)

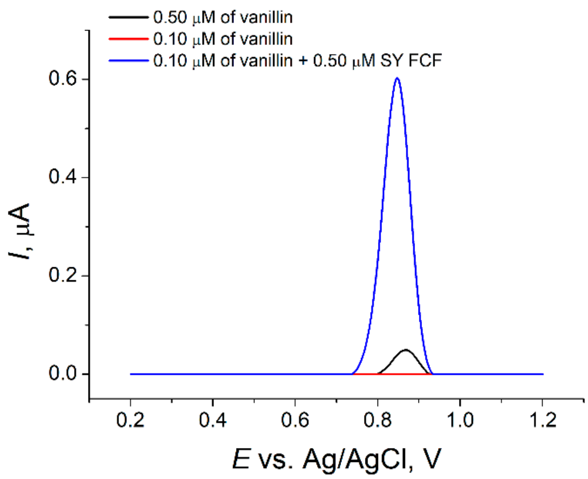

(**b**)

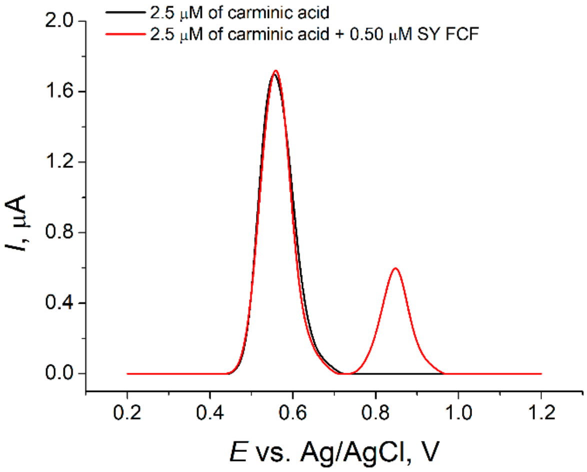

(**c**)

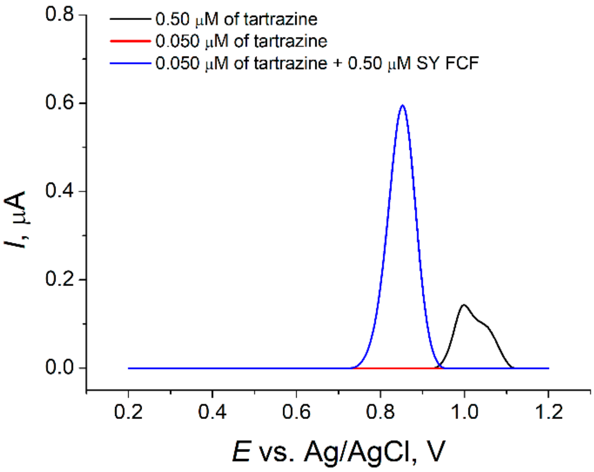

(**d**)

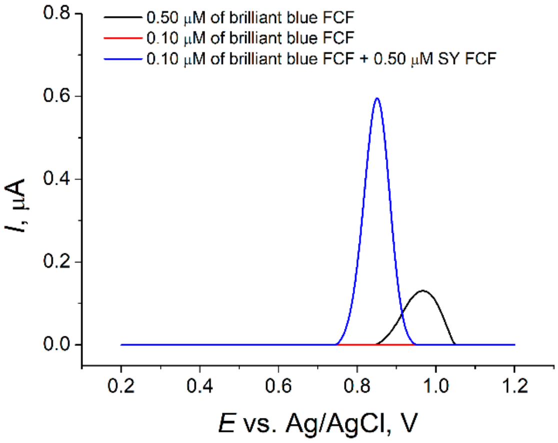

(**e**)

Figure S4. Baseline-corrected differential pulse voltammograms of interferences and their mixtures with SY FCF at the GCE/SnO_2–CeO_2 NPs–HDPB in BRB pH 2.0: (**a**) ascorbic acid; (**b**) vanillin; (**c**) carmnic acid; (**d**) tartrazine; (**e**) brilliant blue FCF. Pulse amplitude is 100 mV, pulse time is 25 ms, potential scan rate is 0.010 V s^{-1}.

Table S1. Recovery of SY FCF in soft drinks (n = 5; P = 0.95).

Sample	Spiked, nM	Found, nM	RSD, %	*R*, %
Sample 1	0	100 ± 5	4.3	
	50	150 ± 6	3.1	100 ± 4
	100	201 ± 4	1.7	101 ± 2
Sample 2	0	176 ± 3	1.2	
	90	266 ± 6	1.9	100 ± 2
	170	345 ± 3	0.55	99.8 ± 0.9
Sample 3	0	31 ± 1	3.5	
	16	47 ± 2	3.1	100 ± 4
	31	62 ± 1	1.4	100 ± 2
